# Intravascular imaging for acute coronary syndrome

**DOI:** 10.1038/s44325-025-00052-y

**Published:** 2025-05-28

**Authors:** Qingyue Tan, Zhiqing Wang, Fan Yang, Sant Kumar, Fu Wang, Jiawen Li, Jigang Wu, Shengxian Tu

**Affiliations:** 1 Biomedical Instrument Institute, School of Biomedical Engineering, Shanghai Jiao Tong University, Shanghai, China; 2 Department of Cardiology, Huadong Hospital, Fudan University, Shanghai, China; 3 Creighton University School of Medicine, Department of Cardiology, Phoenix, Arizona USA; 4 School of Electrical and Mechanical Engineering and Institute for Photonics and Advanced Sensing, University of Adelaide, Adelaide, SA 5005 Australia; 5 Biophotonics Laboratory, University of Michigan-Shanghai Jiao Tong University Joint Institute, Shanghai Jiao Tong University, Shanghai, China; 6 Erasmus University Medical Center, Thoraxcenter, Department of Cardiology, Rotterdam, the Netherlands

**Keywords:** Cardiology, Interventional cardiology

## Abstract

Intravascular imaging is crucial for managing acute coronary syndrome (ACS) by identifying atherothrombotic causes, detecting vulnerable plaques, and guiding percutaneous coronary interventions. Over the past decade, advances in imaging, post-processing, hybrid morphological and molecular techniques, artificial intelligence, and computational modelling have enhanced clinical applications. This review summarizes current intravascular imaging modalities, their utility in ACS patients, and future directions to facilitate their appropriate use in clinical practice.

## Introduction

Acute coronary syndrome (ACS), which includes myocardial infarction and unstable angina, claims the lives of more than 7 million people worldwide each year^1^. Despite substantial diagnostic and therapeutic advances, ACS continues to pose significant health challenges globally, with a high risk of recurrent ischaemic events and cardiac death^1^. Appropriate management and clinical prognosis of ACS patients are largely dependent on the underlying atherothrombotic causes of the culprit lesion, including plaque rupture, erosion, and calcified nodules, thus requiring detailed plaque assessment for accurate decision-making^2,3^. Meanwhile, the highly prevalent complex lesion profile and residual thrombus in situ may further result in more frequent suboptimal stent implantation associated with adverse outcomes^4^. In addition, vulnerable plaques frequently observed in deferred nonculprit lesions can also contribute to the retained high risk of recurrent events in this setting, according to the concept of the pan-vessel phenomenon^5^. Together, these issues complicate ACS management and underscore the imperative need for improved percutaneous coronary intervention (PCI) practices and follow-up outcomes in this setting.

In these regards, intravascular imaging can have critical implications for the detection of culprit lesions, and the identification of vulnerable nonculprit plaques, guiding optimal PCI in the context of ACS by providing valuable insights into plaque composition, vessel size, extent of atherosclerotic disease, as well as stenting results^6,7^. Over the past decade, technological advances and accumulating clinical evidence have significantly expanded the applications of intravascular imaging in ACS. As research progresses, major cardiovascular guidelines have endorsed the use of intravascular imaging for ACS management in the catheterization laboratory^8,9^.

**Fig. 1:**
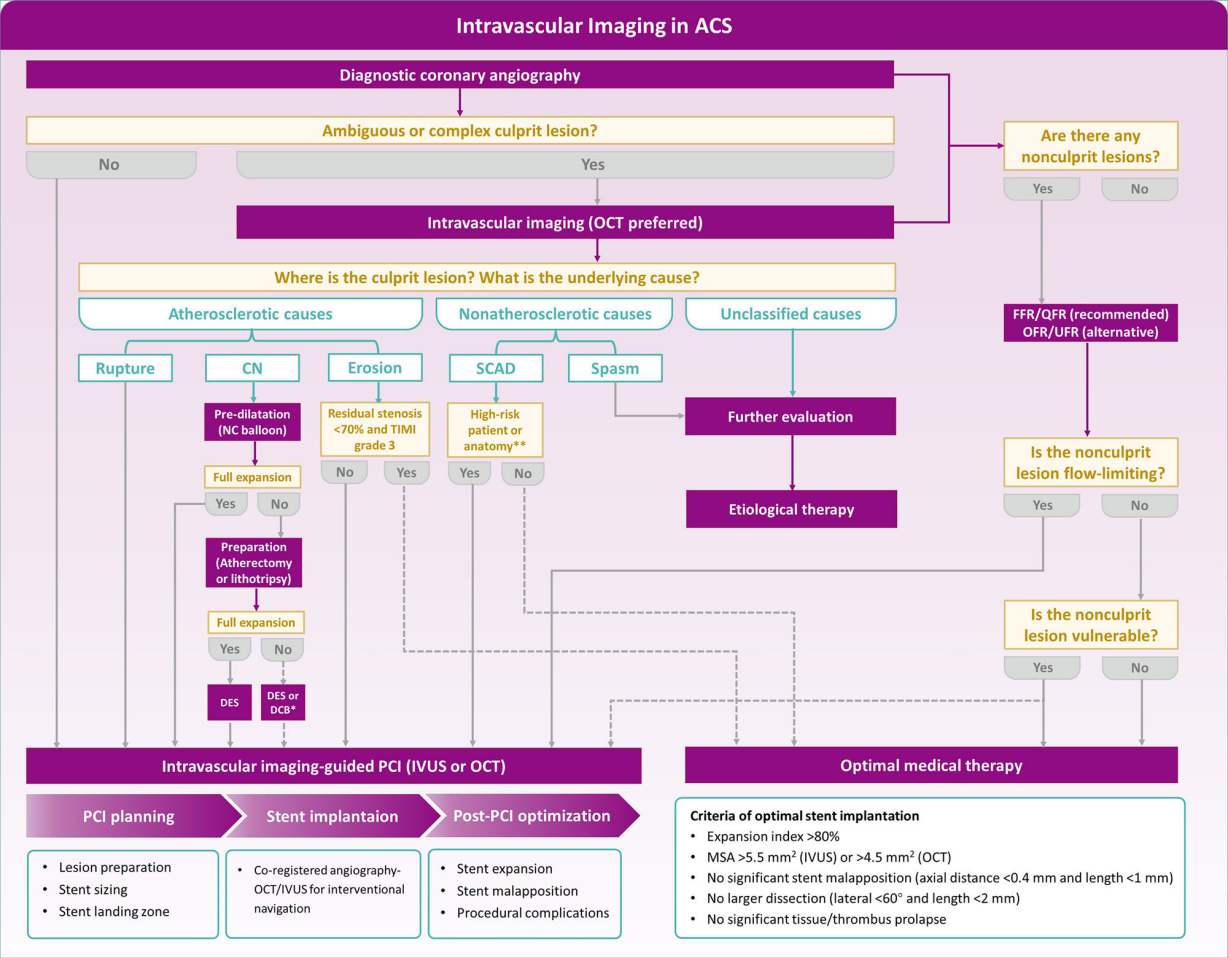
Step-wise approach of performing intravascular imaging in ACS. *The use of DCB instead of DES may be appropriate but remains to be validated by future clinical trials. **High-risk patients indicate those having ongoing ischemia/hemodynamic instability and high-risk anatomy indicates left main or proximal 2-vessel SCAD, dashed lines indicate uncertain or secondary strategies, solid lines indicate more established and recommended strategies. *CN* Calcified Nodules, *TIMI* thrombolysis in myocardial infarction, *SCAD* Spontaneous Coronary Artery Dissection, *NC Balloon* Non-Compliance Balloon, *DES* Drug-Eluting Stent, *DCB* Drug-Coated Balloon.

In this review, we summarize the clinical applications of intravascular imaging in ACS, including evidence from the use of novel imaging modalities and post-processing techniques, and provide a step-wise workflow (Fig. 1) for the appropriate use of intravascular imaging in ACS, outlining key phases from the identification of culprit lesion to PCI guidance.

## Overview of Intravascular Imaging Modalities

Intravascular ultrasound (IVUS) and optical coherence tomography (OCT) represent two widespread intravascular imaging modalities for coronary luminal and plaque morphology assessment with each having its respective pros and cons. By measuring the interference signal and intensity of backscattered light from tissue structures, OCT achieves high-resolution imaging with an axial resolution of 10–20 μm and a lateral resolution of 20–90 μm, much higher than that of IVUS, at the expense of penetration depth (1–2 mm)^10^. In contrast, the technical strengths of IVUS lie in the deep tissue penetration (5–6 mm) and no need of blood clearance, despite a limited resolution (axial 20–100 μm, lateral 150–250 μm). Significantly, the integration of IVUS and OCT to overcome the limitations of each technique can be achieved through the dual-probe catheter design for synchronous image acquisition^11^. Near-infrared spectroscopy (NIRS) uses near-infrared light waves (800–2500 nm) to illuminate the coronary artery wall and has the unique advantage for the detection of lipid content. Even so, NIRS lacks depth resolution and cannot provide detailed information about tissue structures. The hybrid system combining NIRS with IVUS or OCT to offer complementary plaque morphology and composition information thus has been a key development focus in recent years^12,13^. Currently, IVUS-OCT, IVUS-NIRS, and OCT-NIRS imaging system are commercially available with favorable safety profiles and clinical utility^11,14,15^. Despite an overall limited clinical penetration^16^, intravascular imaging has been increasingly used during the past decade, coupled with a growing body of evidence supporting the beneficial roles in ACS. In addition, novel molecular imaging techniques (e.g., near-infrared fluorescence [NIRF], fluorescence lifetime imaging [FLIm], and intravascular photoacoustic imaging [IVPA]) and hybrid imaging systems have emerged as promising modalities for clinical use^17,18^. Artificial intelligence (AI) and computational modelling have also been incorporated into the analytical procedure to improve and extend intravascular imaging assessment by facilitating image processing and evaluations of coronary physiology and biomechanics, respectively^19–21^. Recent attempts to apply these novel modalities and post-processing techniques to preclinical/clinical studies have been successful in enhancing the accuracy and efficacy of intravascular imaging. Comparison of the technical characteristics and clinical utilities among standalone intravascular imaging modalities is presented (Table 1).

**Table 1:** Comparison of different standalone intravascular imaging modalities

	IVUS (<60 MHz)	OCT	NIRS	NIRF	FLIm	IVPA
Technical features						
Blood clearance	NO	YES	NO	YES	YES	YES
Axial resolution	20–100 μm	10–20 μm	NA	NA	NA	20–80 μm
Lateral resolution	100–250 μm	20–90 μm	NA	NA	10–30 μm	100–250 μm
Penetration depth	>5 mm	1–2 mm	~1 mm	~0.5 mm	<150 μm	3–4 mm
Imaging speed	30 fps	100-200 fps	30 fps	>50 fps	~10 fps	~20 fps
Anatomic information	YES	YES	NO	NO	NO	YES
Commercial availability	YES	YES	YES	NO	NO	NO
Culprit lesion						
Plaque rupture	++	+++	-	-	-	-
Plaque erosion	+ (HD- IVUS)	+++	+	+	-	-
Calcified nodules	++	+++	-	-	-	-
Spontaneous dissection	++	+++	-	-	-	-
Thrombus	++	+++	-	-	-	-
Nonculprit lesion						
Plaque burden	+++	+/++ (with AI)	-	-	-	-
Vessel remodelling	++	-	-	-	-	-
Fibrous cap thickness	+	+++	-	-	-	-
Lipid component	+	++	+++	-	-	-
Calcium thickness	-	+++	-	-	-	-
Detection of deep calcium	+++	-	-	-	-	-
Myocardial bridge	++	+	-	-	-	-
Inflammation	-	++	-	++	+++	-
Pre-PCI assessment						
Stent diameter sizing	+++	+++	-	-	-	-
Stent length sizing	+++	+++	-	-	-	-
Post-PCI assessment						
Stent expansion/malapposition	++	+++	-	-	-	-
Tissue/thrombus protrusion	++	+++	-	-	-	-
Stent edge dissection	++	+++	-	-	-	-
Stent failure assessment						
In-stent restenosis	++	+++	-	-	-	-
Stent thrombosis	++	+++	-	-	-	-

-poor, +feasible, ++good, +++excellent. *HD-IVUS* High-Definition Intravascular Ultrasound, *NA* Not Applicable.

## Identification of Culprit Lesions

**Fig. 2:**
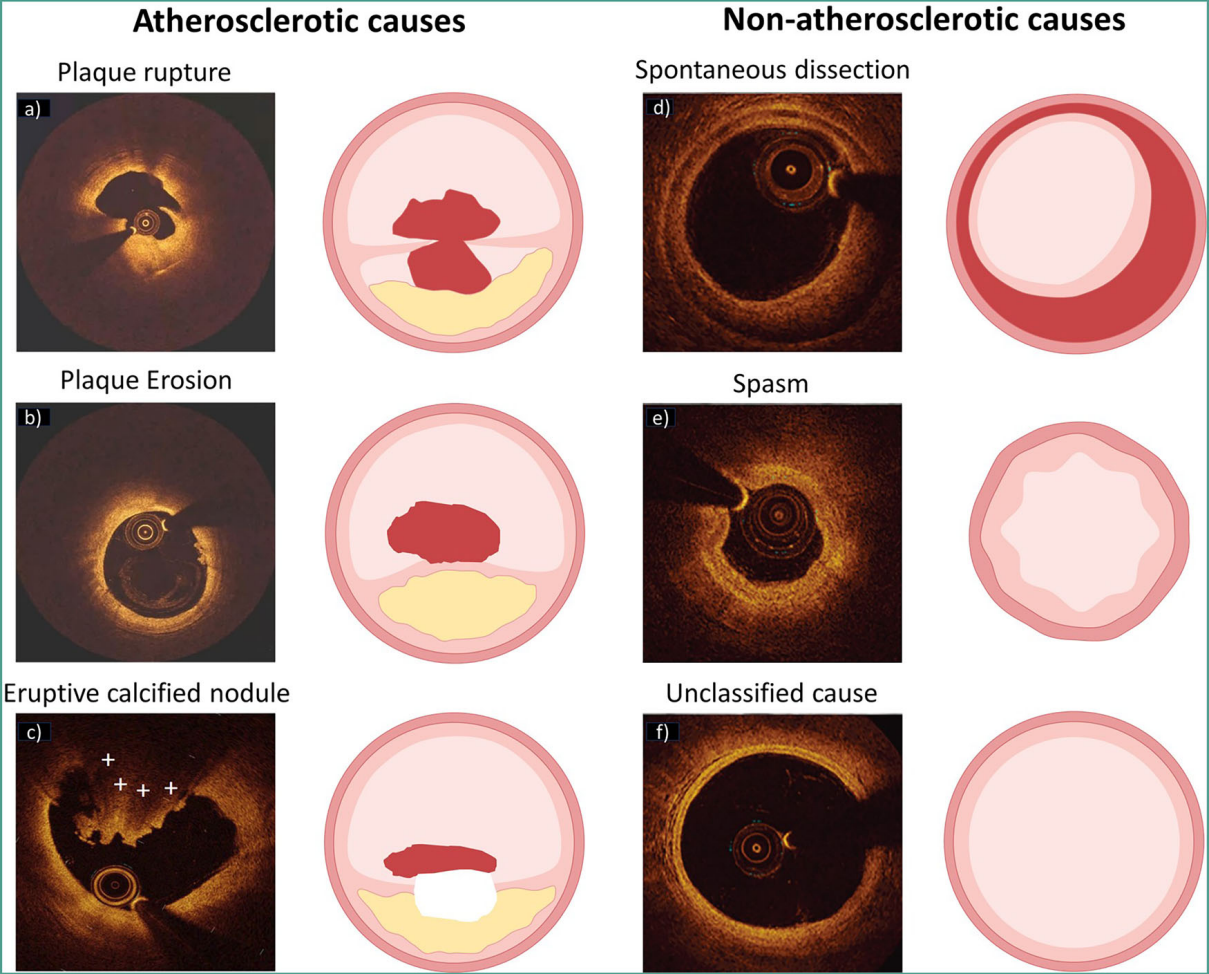
OCT imaging features of atherosclerotic and nonatherosclerotic coronary artery lesions. Figure **a-b** acquired by the core laboratory. Figure **c** adapted from Sugiura et al.^151^. Figure **d**-**f** adapted from Zeng et al.^31^ Copyright © (2023), with permission from the American College of Cardiology Foundation.

Historically, the mechanism of atherothrombosis was thought to be primarily associated with the rupture of a vulnerable plaque. However, histopathological studies subsequently demonstrated that plaque rupture only accounted for 60−65% cases of ACS, while 30−35% and ~5% cases were found to be attributed to plaque erosion and eruptive calcified nodules, respectively (Fig. 2)^22,23^. Identifying culprit lesions in ACS remains a cornerstone of effective management, yet diagnostic angiography often fails to pinpoint the location of the culprit lesion, let alone the recognition of the underlying cause or the exclusion of nonatherosclerotic causes. In a large-scale study including 4793 patients presenting with ST-elevation ACS triaged for immediate angiography, nonobstructive ( < 50% stenosis) and normal coronary artery were found in 5% and 6% cases, respectively, and had even worse outcomes as compared with those having obstructive coronary stenosis^24^. For patients presenting with non-ST-elevation ACS, nonobstructive coronary artery disease could be found in over 30% cases^25^. In the past decades, the advent of intravascular imaging has provided the opportunity to allow in vivo visualisation of culprit lesion location and underlying atherothrombotic causes in ACS, especially in case of angiographically ambiguous findings and myocardial infarction with nonobstructive coronary disease.

In particular, OCT is generally preferred in this context owing to its high resolution. Plaque rupture on OCT is generally characterized by a disrupted fibrous cap with a cavity inside a lipidic plaque^26^. Since clinically available OCT is still insufficient to visualize the endothelial cells, the definition of plaque erosion is mainly based on indirect features, which is typically characterized by an intact fibrous cap with overlying thrombus or irregular luminal surface in the absence of thrombus^27^. However, detection of definite plaque erosion is not always available since the overlying thrombus may have been dissolved before OCT imaging, thus requiring the comprehensive consideration of the clinical data. Notably, a transformer-based deep learning (DL) model has been recently trained to facilitate automatic detection of plaque erosion on OCT and showed a higher accuracy than convolutional neural network (area under the curve, 0.91 vs. 0.84) with expert readers as reference standard^28^. As a less common cause of ACS, calcified nodule is currently described as an eruptive calcific nodule protruding into the lumen with fibrous cap disruption^27^. Thrombus secondary to the aforementioned causes typically appears as an intramural mass attached to the luminal surface or floating within the coronary lumen, with high backscattering and attenuation for a red thrombus and with homogeneous backscattering and low attenuation for a white thrombus. Fortunately, the complicated features of thrombus on OCT can now be automatically segmented using a DL model with high efficiency and accuracy^29^. Furthermore, OCT can also be helpful to identify infrequent non-atherosclerotic causes of ACS, such as spontaneous coronary artery dissection and coronary spasm^30,31^. A recent study assessing the causes underlying myocardial infarction with nonobstructive coronary artery by OCT identified a non-atherosclerotic cause in 61.1% patients, with 4.2% being spontaneous dissection and 4.7% being coronary spasm^31^.

Despite the utility for the visualisation of plaque rupture and calcified nodules^6,32^, conventional IVUS imaging (<60-MHz) often fails to identify plaque erosion and shows a low sensitivity (57%) to detected intraluminal thrombus^33^. IVUS is also less effective than OCT in reliably distinguishing intramural hematoma, a typical case of spontaneous coronary artery dissection, from lipid-rich plaque due to their similar appearance as hypoechoic areas in the vessel wall^34^. However, with sustained technical advances, IVUS may also hold the promise to discriminate atherothrombotic causes, albeit less pronounced than OCT^35,36^. High-definition IVUS with a 60-MHz transducer has recently been demonstrated with the potential of direct visualisation of plaque erosion compared with OCT in a series of patients, but required to be extensively validated^37^. In a recent study using an IVUS-NIRS hybrid imaging system, evaluation of indirect imaging signs, including plaque cavity, convex calcium, and maximum lipid core burden index in any 4-mm longitudinal segments (maxLCBI_4mm_) showed high sensitivity and specificity to differentiate plaque rupture, plaque erosion, and calcified nodule^38^. Considering the requirement of blood clearance for OCT imaging which is not so straightforward especially in the setting of primary PCI, IVUS-NIRS or high-definition IVUS may be qualified as a surrogate for culprit lesion assessment.

Importantly, different ACS causes have been associated with distinct plaque characteristics and risks of recurrent events. Culprit lesions with plaque rupture frequently have a more vulnerable appearance as compared with those having intact fibrous cap, including plaque erosion and calcified nodule^12^. Of these causes, real-world ACS studies have shown that patients with intact fibrous cap generally have a smaller infarct size with better clinical outcomes than those with plaque rupture^39,40^. In a recent study, Gerhardt et al. ^41^ observed that ACS patients with intact fibrous cap for culprit lesions on OCT had much lower plasma levels of systemic inflammatory biomarkers and a lower risk of recurrent cardiac events compared with those having plaque rupture. In another study further distinguishing calcified nodule from plaque erosion, Kondo et al. ^42^ reported that calcified nodule, despite being the least common, was associated with the highest risk of recurrent events, followed by plaque rupture and plaque erosion. This finding might be explained by the relatively older age, more comorbidities, and more severe atherosclerosis in patients with calcified nodule, thus predisposing to a higher risk of events^43^.

Evidence from intravascular imaging enables a better understanding of atherothrombosis underlying ACS and should have critical implications for future paradigm shift in the management of ACS. Although revascularization of a culprit lesions is generally recommended to minimize the risk of recurrent events^44,45^, additional lesion preparation with atherectomy or intravascular lithotripsy or their combination before stenting is required according to the pre-dilatation using a noncompliant balloon, when a calcified nodule is identified^43^. After that, implantation of drug-eluting stents is clinically recommended. Notably, drug-coated balloons may be appropriate for selected cases with partial expansion following lesion preparation and the absence of major dissections to avoid subsequent stent failure and warrant further investigation^43^. In contrast, the EROSION trial has demonstrated the possibility of medication alone for selected ACS patients with plaque erosion (residual diameter stenosis <70% and Thrombolysis in Myocardial Infarction flow grade 3)^46^. A conservative treatment strategy may also be advocated for spontaneous coronary dissection unless having ongoing ischemia/hemodynamic instability or high-risk anatomy (left main or proximal 2-vessel)^47^, since most spontaneous coronary dissection lesions may heal over time and PCI for these lesions is generally associated with a high incidence of procedural complications^47,48^.

## Detection of Vulnerable Plaques in Nonculprit Lesions

Data from intravascular imaging studies have revealed that nonculprit lesions in ACS patients with multivessel disease have more vulnerable plaque characteristics when compared with non-ACS patients, resulting in worse outcomes and less pronounced benefits from coronary physiology-guided revascularisation^5,49^. Early discrimination of these vulnerable nonculprit lesions is therefore of critical importance for optimal management and improved outcomes.

Attenuated plaque detected by grayscale IVUS, defined as backward signal attenuation without obvious calcification, is a vulnerable plaque indicator which is more common in ACS patients than in those with stable angina^50^. Validation studies demonstrated that attenuated plaque generally coexisted with other vulnerable characteristics at least including large necrotic core and thin-cap fibroatheroma (TCFA), and was likely to result in no-reflow after PCI and an increased risk of future adverse events^50–52^. In the PROSPECT study specific for ACS patients, virtual histology IVUS-derived vulnerable characteristics, including plaque burden (PB) >70%, positive remodelling, as well as TCFA were all found to be predictive of Major Adverse Cardiac Events (MACE) during mid-term follow-up^53,54^. Although virtual histology IVUS is promising, it is underpowered to detect fibrous cap thickness and lipid-rich plaque (LRP) and the definition of TCFA by virtual histology IVUS is not exactly the same as that by histopathology^55^. For calcium detection, although IVUS showed a higher sensitivity than angiography^56^, the existing of acoustic shadowing, reverberation, and non-uniform rotational distortion might frequently diminish the accurate quantification of calcified plaques, especially the assessment of calcium thickness^57^.

By contrast, OCT provides more detailed visualisation of plaque morphology in particular fibrous cap and microstructures, enabling the detection of multiple vulnerable plaque features, like circumferential extent of lipid pool, fibrous cap thickness, and inflammatory content. The Massachusetts General Hospital OCT registry based on an all-comer population with 39.6% being ACS demonstrated that the presence of OCT-detected LRP in the nonculprit regions of the target vessel, defined as a lipid arc of greater than one quadrant, was independently associated with development of nonculprit-MACE events^58^. In the CLIMA study enrolling 1003 patients (53.4% ACS) with angiographically nonobstructive lesions, comprehensive OCT analysis identified four plaque vulnerability features (minimal lumen area <3.5 mm^2^, fibrous cap thickness <75 μm, lipid arc >180°, and OCT-defined macrophages) to be predictive of future MACE events^59^. This study identified OCT-detected vulnerable plaque, TCFA, with all fibrous cap thickness, lipid arc, and local inflammation being taken into consideration, as the strongest predictor of follow-up events and the simultaneous presence of all the four features was associated with an even higher risk of events (hazard ratio=7.54, *P* < 0.01). The robust correlation of TCFA with ACS was further confirmed in another in vivo OCT study focusing on extremely high-risk patients with recurrent ACS^60^. However, another vulnerable plaque feature, healed plaque, defined as a heterogenous, layered plaque suggestive of previous rupture or erosion and subsequent healing and growth^61^, was rarely detected in patients with recurrent ACS, but presented in 28.9% patients with long-standing stable angina pectoris. Future studies are needed to provide a mechanistic understanding of the role of healed plaque in atherosclerosis and the onset of ACS. Recently, a novel vulnerable plaque indicator, called lipid-to-cap ratio (LCR), was proposed by integrating cap thickness and the specific lipidic content instead of the lipid arc, with AI software enabling the automatic calculation of LCR for greater objectivity and reliability^62,63^. In nonculprit lesions of ACS patients, LCR > 0.33 showed more profound prognostic significance than any other morphological parameter including TCFA and the combination of optical flow ratio (OFR) ≤ 0.84 and LCR > 0.33 enabled more accurate prediction of future adverse events^62^. Moreover, unlike IVUS, OCT is capable of assessing calcium thickness, thus enabling the quantification of calcium area and volume. However, the assessment could be underestimated in deeper calcium due to the superficial plaque attenuation^64^.

Notably, the morphology of nonculprit lesions might also vary substantially according to the atherothrombotic cause underlying ACS. Studies with 3-vessel OCT imaging for pancoronary arteries have demonstrated that culprit lesions with plaque rupture generally had more profound pancoronary vulnerability as compared with those with plaque erosion, including macrophage accumulation, microvessel, and spotty calcium^65–67^. The distinct pancoronary vulnerability patterns might partially explain the prognostic difference between ACS patients with plaque rupture and erosion.

NIRS enables direct and accurate quantification of lipid content to detect LRP with prognostic significance^68,69^. In the PROSPECT II study, 902 high-risk patients with recent myocardial infraction were prospectively recruited and underwent 3-vessel imaging with a hybrid IVUS-NIRS system^14^. During a median follow-up of 3.7 years, patients with at least one untreated nonculprit lesion with maxLCBI_4mm_ ≥324.7 on NIRS had an increased risk for non-culprit lesion-related adverse outcomes (odds ratio=2.27, P = 0.007). In addition, the incorporation of maxLCBI_4mm_ ≥324.7 provided incremental prognostic value over PB ≥ 70%. In a comparative study of IVUS-NIRS and OCT for identifying LRP, Vito et al. ^70^ reported only a mild correlation of lipid arc between these two modalities. However, findings of this study could not conclude which modality was superior, since comparison with histopathology was lacking.

However, most morphological vulnerable plaques seem to stabilize over time and do not eventually cause an acute coronary event, whereas some others together with thick-cap fibroatheromas may progress rapidly under the complex interplay between unrecognized systemic and local proatherogenic factors, thus highlighting the necessity of more precise and comprehensive plaque evaluation^71^. In this regard, one solution should lie in the technical iterations for the conventional intravascular imaging modalities, such as high-/dual-frequency transducer IVUS, all-optical intravascular ultrasound, and to achieve high-resolution or in-depth penetration or both^72–74^. Novel molecular imaging modalities like NIRF/FLIm can provide valuable insights into the molecular signatures of plaque vulnerability^75,76^. Recent studies have confirmed that near-infrared autofluorescence is associated with the presence of oxidative stress and intraplaque hemorrhage^77,78^. With the administration of indocyanine green, an in vivo study showed that NIRF signal-enhanced regions generally co-localized with macrophage-abundant and lipid-rich areas^79^. FLIm may allow the quantification of inflammatory activity and the discrimination of major coronary plaque components, with the assist of DL models^75,76^. In-human study testing the utility of integration of NIRF/FLIm with IVUS/OCT for hybrid imaging is ongoing and the results are anticipated (NCT04835467).

The limited predictive strength based on plaque morphology alone also facilitated the incorporation of biomechanical forces into intravascular imaging-based evaluation to generate synergetic effects on risk stratification^80^. Low wall shear stress is thought to be a critical local factor associated with advanced lesion, while plaque structural stress is highly dependent on plaque composition and directly results in plaque rupture when increased plaque structural stress ( >300 kPa) exceeds fibrous cap strength^81–83^. Further advances in intravascular imaging and computational modelling are expected to improve the methodological feasibility of biomechanical simulation for a wider clinical application.

Whether revascularisation beyond optimal medical treatment for vulnerable plaques is beneficial remains a matter of an ongoing dispute and has received growing attention in recent years. The PREVENT trial was designed to test whether preventive PCI of non-flow-limiting vulnerable plaques could reduce 2-year MACE events in an all-comer population and demonstrated an absolute risk reduction of the primary endpoint events by 3.0% (0.4% vs. 3.4%, hazard ratio=0.54, P = 0.0097)^84^. Two studies assessing PCI for intravascular imaging-detected vulnerable plaques in nonculprit lesions in ACS patients are underway (VULNERABLE [NCT05599061] and INTERCLIMA [NCT05027984]).

## Guidance of PCI Planning

Coronary calcification represents a major determinant of stent underexpansion^17^. For fibrous or lipid-rich plaques, pre-dilatation with a compliant or semi-compliant balloon or direct stent deployment is adequate. Conversely, the presence of moderate to severe calcification typically requires appropriate lesion preparation prior to stent implantation through specific techniques, like pre-dilatation with a cutting or scoring balloon, intravascular lithotripsy, rotational atherectomy, or shock wave therapy^85^. Calcified plaque causes acoustic shadowing on IVUS, thus diminishing accurate quantification of thickness; by contrast, near-infrared light can easily penetrate calcified tissue, enabling OCT as a unique technique for quantifying calcium plaques. According to a proposed OCT-based calcium scoring system, calcified plaques with maximum angle >180°, maximum thickness >0.5 mm, and length >5 mm are at the highest risk of stent underexpansion and plaque modification prior to stent implantation is strongly recommended^85^. Additionally, IVUS-detected attenuated plaque has been found to predictive of no-reflow immediately after stent implantation in ACS patients undergoing primary PCI^50^. In a randomized study enrolling 200 ACS patients having attenuated plaque with longitudinal length ≥5 mm on IVUS, PCI with distal protection was associated with a lower incidence of no-reflow and fewer adverse cardiac events after PCI when compared with conventional treatment^86^. Notably, AI could assist to accurately predict PCI results based on pre-procedural intravascular imaging. Using a convolutional neural network, Min et al.^87^ developed an effective model integrating pre-procedural IVUS images and clinical information for predicting stent underexpansion, revealing an excellent correlation between predicted and actual minimal stent area (r = 0.832) and total stent volume (r = 0.958).

Methodological differences exist between IVUS and OCT, as IVUS generally overestimates the lumen area by ~10% while OCT has relatively low penetration for delineation of the external elastic membrane^88^. These differences, however, did not translate into significant disparities in angiographic findings immediate after PCI or clinical outcomes during follow-up^89^. A recent Consensus Document advocated a feasible algorithm for the selection of stent diameter using either IVUS or OCT: (1) Distal lumen reference based sizing with subsequent optimization of the mid and proximal stent segments; (2) Mean reference lumen diameter (average of proximal and distal) with up rounding stent 0–0.25 mm; (3) Mean external elastic membrane diameter with down rounding to the nearest 0.25 mm stent size^90^. The selection of stent length and landing zone should ensure the full coverage of the lesion segment with residual PB <50% and no lipid-rich tissue at the stent edge. In a recent study, an OFR-based virtual stenting technique simulating the effect of stent implantation in the target lesion segment from pre-PCI OCT images was developed to assist the planning process of stent placement^91^. The simulated residual OFR holds promise to help select the best stent diameter, length, and landing zone to obtain the maximally achievable post-PCI physiological results beyond optimal morphological stenting outcomes and may deserve further attention.

Notably, off-line coregistration of angiography and intravascular imaging has been successfully achieved with the assist of Al algorithms^92^. Incorporation of intravascular imaging to angiography in a catheterization laboratory should be promising for procedural navigation by providing real-time and 3D visualisation of the coronary lumen morphology, lesion location, together with plaque composition, thus enabling more precise stent placement.

## Guidance of PCI Optimization

The increasing use of intravascular imaging has identified multiple suboptimal stenting results. Stent underexpansion, assessed by either absolute (minimal stent area [MSA]) or relative (stent area/reference area) parameters, is established as a key predictor of stent failure. However, the definitions of absolute and relative stent underexpansion may vary significantly between different studies and between IVUS and OCT. The CLI-OPCI trial identified OCT stent underexpansion according to MSA <4.5 mm^2^ as an independent predictor of MACE^93^. In a pooled analysis of multiple IVUS studies, post-PCI MSA was predictive of 9-month follow-up stent patency with an optimal threshold of <5.7 mm^2^ and <6.4 mm^2^ for paclitaxel-eluting stent and bare-metal stent, respectively^94^. In the drug-eluting stent era, a MSA of > 4.5 mm^2^ by OCT or >5.5 mm^2^ by IVUS is currently recommended for non-left main coronary artery disease to achieve optimal stent expansion^90^. By contrast, there is currently a lack of uniform criteria with respect to relative stent underexpansion. In a substudy of the ADAPT-DES registry, Fujimura et al. ^95^ compared a number of relative stent expansion indices based on different calculation methods and distinct reference lumen definitions. They observed that only stent/vessel area at the MSA site ≤38.9% was independently associated with 2-year clinically driven target lesion revascularisation or definite stent thrombosis. However, the majority of other studies adopted the average area of the proximal and distal reference lumen to calculate the expansion index with a threshold of either >80% or >90% as the criterion of optimal stent expansion. Considering that expansion index >90% could hardly be achieved in most cases, a threshold of >80% was finally recommended^90^. In addition, although sufficient stent expansion is required, excessive expansion is likely to result in vessel wall injury and stent strut fracture^96,97^. Since no criteria regarding the upper limit of the range of relative stent expansion are currently available, more attention should be paid on this issue in future studies.

Stent malapposition refers to the separation of stent struts from the intimal surface of the vessel wall and can be categorized as acute and late acquired malapposition. Acute stent malapposition, presumably due to undersized stent implantation, stent underexpansion, or intra-stent aneurysm/ectasia in situ, is highly prevalent on OCT (39.1−72.3%) than on IVUS (7.3 − 38.5%)^98^. Of note, most acute stent malapposition may resolve overtime, resulting in an indefinite correlation with follow-up stent failure^99,100^. According to the OCT study by Lee et al.^99^, acute stent malapposition with axial distance <0.4 mm and longitudinal length <1 mm should be acceptable^90^. Late−acquired malapposition during follow-up is generally supposed to be associated with de inflammatory process and positive vessel remodelling and been recognized as a risk factor of very late stent thrombosis^101,102^.

Suboptimal stenting results also include tissue/thrombus protrusion and geographical miss (e.g., residual PB > 50% at stent edge, stent edge dissection/hematoma). In particular, tissue/thrombus protrusion is more frequently observed in ACS patients, but its clinical significance remains controversial. In the ADAPT-DES study enrolling all-comer patients, IVUS-detected tissue protrusion after stent implantation was not associated with adverse outcomes^103^. Nevertheless, the CLI-OPCI study targeting ACS patients demonstrated a significant association between tissue/thrombus protrusion, defined as tissue ≥500 μm in thickness prolapsing into the vessel lumen, with device-oriented cardiovascular events^4^. Another study further classified tissue protrusion into 3 different types (smooth protrusion, disrupted fibrous tissue protrusion, and irregular protrusion), only irregular protrusion, which occurred in 53.8% cases, had prognostic significance^104^. It is speculated that the inconsistent findings regarding the prognostic implications of tissue/thrombus protrusion might be attributable to the different study populations and imaging modalities. Dissection, a type of geographical miss, frequently occurs at the distal stent edge, mainly because of the aggressive stent dilatation, a tapered vessel morphology, and the existence of calcium and attenuated plaque at site. Large dissections with extensive lateral >60° and longitudinal length >2 mm on IVUS or OCT are considered to be suboptimal and required for further treatment^4,90^. In addition, the use of computational coronary physiology for post-PCI assessment can provide incremental prognostic value over intravascular imaging alone and the combination of them has emerged as an area of interest, in particular with coronary physiology concurrently calculated from intravascular imaging pullback^105^. A recent study using combined OCT and OFR for post-PCI assessment in ACS patients demonstrated a prevalence of suboptimal stent implantation in 50.2% patients, with stent underexpansion (expansion index <80%), MSA <4.5 mm^2^, stent edge lipid-rich plaque (lipid arc of >90°), and OFR < 0.90 as independent predictors of target lesion failure at 1 year, and a significant improvement of the reclassification for target lesion failure with their combination^106^. Future prospective, high-volume studies are thereby necessary to further validate the prognostic significance of post-procedural intravascular imaging-derived coronary physiology assessment, especially when combined with morphological stenting results.

The clinical benefits of image-guided drug-eluting stent implantation have been demonstrated in several meta-analyses. In the recent network meta-analysis including 22 trials, intravascular imaging-guided PCI showed significant superiority over angiographic guidance with respect to the safety and effectiveness, with an overall risk reduction by 29% for target lesion failure, and the utility was similar between IVUS and OCT^107^. Of which, several trials included in this meta-analysis, for instance, OCTACS^108^, DOCTORS^109^, and a small-scale randomized control trial by Kala et al. ^110^, were designed specialized for ACS patients (Table 2). In these studies, intravascular imaging (OCT) was reported to be associated with improved stenting results immediately after PCI as well as better strut coverage and less MACE events during short-term follow-up, without significantly increasing periprocedural complications. Recently, emerging evidence have further indicated the beneficial role of intravascular imaging in guiding optimal PCI. In the large-scale IVUS-ACS Trial, 3505 ACS patients were 1:1 randomized to IVUS- or angiography-guided PCI^7^. The criteria for IVUS-guided optimal PCI included MSA > 5.0 mm^2^ or relative stent expansion >80% (MSA divided by distal/proximal reference lumen area), residual PB < 55% within 5 mm proximal or distal to the stent edge, and the absence of dissection over 3 mm in length. During 1-year follow-up, the incidence of the primary endpoint, target vessel failure, was 4.0% in the IVUS-guided group versus 7.3% in the angiography-guided group, resulting in a 45% risk reduction. The OPINION ACS Trial comparing OCT and IVUS-guided PCI strategy in ACS patients demonstrated the non-inferiority of OCT with comparable in-stent minimal lumen area at 8 months^111^. Several other retrospective studies have further confirmed the findings regarding the utility of IVUS-guided PCI and the equivalence between IVUS and OCT in the context of acute myocardial infarction^112–115^. In particular, ACS patients with ostial left main stenosis and chronic kidney disease often having moderate to severe calcification lesions were expected to benefit more from IVUS guidance^112,114^. Therefore, despite the overall comparable benefits between IVUS and OCT for PCI guidance, IVUS is generally preferred in several special conditions, including ostial left main stenosis and patients with chronic kidney disease, largely due to the difficulty of complete blood clearance in the aorto-ostial segment and increased dosage of contrast medium^116,117^.

**Table 2:** Summary of clinical trials investigating the benefits of intravascular imaging-guided PCI in ACS patients

Trial	Year	Study Design	Study Population	Comparison	IVI criteria for optimal PCI	Follow-up	Primary endpoint and results
OCTACS^108^	2015	RCT	NSTEMI	OCT (n = 40) vs. Angio (n = 45)	• Relative stent expansion of >90% (MSA divided by distal/proximal reference lumen area) • No malapposition with ≥3 struts per cross-sectional area with axial distance >140 μm • No stent edge dissection (causing a MLA < 4 mm^2^) • Residual stenosis (causing a MLA < 4 mm^2^)	6-month OCT follow-up	• Strut coverage (1) Percentage of uncovered struts: 4.3% vs. 9.0%, p < 0.01 (2) Percentage of completely covered stents: 17.5% vs. 2.2%, p = 0.02
DOCTORS^109^	2016	RCT	NSTE-ACS	OCT (n = 120) vs. Angio (n = 120)	• Relative stent expansion of >90% (MSA divided by distal/proximal reference lumen area) • No stent edge dissection	NA	• Post-PCI FFR: 0.94 ± 0.04 versus 0.92 ± 0.05, P = 0.005
Kala et al.^110^	2018	RCT	STEMI	OCT (n = 105) vs. Angio (n = 96)	• Relative stent expansion of >80% (MSA divided by average reference lumen area) or >90% (MSA divided by distal reference lumen area) • No significant malapposition • No stent edge dissection	9-month OCT and clinical follow-up	• OCT-detected in-stent area stenosis: 6% vs. 18%, p = 0.0002 • MACE: 3% vs. 2%, p = 0.87
COREA-AMI^112^	2021	Retro	ACS	IVUS (n = 2,032) vs. Angio (n = 7,814)	No dedicated criteria for IVUS-guided PCI	4-year follow-up	• MACE: 15.3% vs. 19.0%, p < 0.001
KAMIR-NIH^113^	2022	Retro	ACS	IVUS (n = 1,887) vs. Angio (n = 7,120)	No dedicated criteria for IVUS-guided PCI	3-year follow-up	• Target lesion failure: 4.8% vs. 8.0%, p < 0.001
KAMIR-NIH^114^	2023	Retro	ACS	IVUS (n = 879) vs. Angio (n = 3,191)	No dedicated criteria for IVUS-guided PCI	3-year follow-up	• Target lesion failure: 6.7% vs. 12.0%, p < 0.001
IVUS-ACS^7^	2024	RCT	ACS	IVUS (n = 105) vs. Angio (n = 96)	For non-left main coronary arteries: • MSA > 5.0 mm^2^ or relative stent expansion of >80% (MSA divided by distal reference lumen area) • Residual PB < 55% within 5 mm proximal or distal to the stent edge • The absence of dissection over 3 mm in length For left main coronary arteries: • MSA > 10 mm² for the left main segment, > 7 mm² for the ostial or proximal LAD, and > 6 mm² for the ostial or proximal LCx	1-year clinical follow-up	• Target vessel failure: 4.0% vs. 7.3%, p = 0.0001
KAMIR-NIH and KAMIR-V^115^	2024	Retro	ACS	OCT (n = 535) vs. IVUS (n = 4,725)	No dedicated criteria for imaging-guided PCI	1-year follow-up	• Target lesion failure: 2.1% vs. 3.4%, p = 0.11
OPINION ACS^111^	2024	RCT	ACS	OCT (n = 70) vs. IVUS (n = 69)	• Relative stent expansion of >80% (MSA divided by the mean reference lumen area) • Incomplete stent apposition	8-month OCT follow-up	•In-stent MLA: 4.91 (4.53-5.30) mm^2^ vs. 4.76 (4.35-5.17) mm^2^, p for non-inferiority <0.001

*RCT* Randomized Controlled Trial, *Retro* Retrospective Study, *STEMI* ST-Elevation Myocardial Infarction, *MLA* Minimal Lumen Area, *LAD* Left Anterior Descending artery, *LCx* Left Circumflex artery, *FFR* Fractional Flow Reserve.

Regardless of the intravascular imaging-based criteria for optimal stent implantation having been recommended by the Consensus Document, clinical studies continue to be conducted following inconsistent standards, leading to the difficulty to properly evaluate these studies and assist clinical practice. Future efforts are needed to establish standard procedures for intravascular imaging-guided lesion assessment and PCI guidance, especially with the use of advanced imaging modalities and the AI-powered image post-processing techniques.

## Evaluation of Stent Failure

In-stent restenosis (ISR) is the most frequent cause of stent failure mainly characterized by neointimal hyperplasia and neoatherosclerosis, with heterogeneous underlying mechanisms involving patient clinical determinants, lesion anatomic characteristics, and procedural and stent-related factors^118^. In the second-generation drug-eluting stent era, the incidence of ISR-related ischemia-driven target lesion revascularisation is estimated to be 2% per year^119^, yet the absolute patient number is considerable due to a large and ever-growing PCI population. Patients with ISR undergoing PCI present more frequently as ACS (51.8% vs. 38.6% in non-ISR patients), with 25% being acute myocardial infarction^120^. ISR can be classified as focal, multifocal, and diffuse on IVUS according to minimal lumen area, involved length, as well as the distribution patterns^121^. In general, OCT is superior to IVUS in terms of the visualisation of stent strut coverage and neoatherosclerosis. In patients with angiographically documented ISR, Gonzalo et al. ^122^ raised a systematic approach to identify differential patterns of ISR on OCT based on multiple aspects, including tissue structure (homogeneous, heterogeneous, or layered type), tissue backscatter, microvessel, lumen shape, and intraluminal material suggestive of thrombus. The morphological assessment using OCT can be informative for the selection of the favorable treatment strategy for each ISR pattern^123^. Accordingly, a workflow for intravascular imaging-guided optimal management of ISR was proposed based on the underlying mechanism and substrate^118^.

Stent thrombosis, including early (<30 days) and late (30 days to 1 year)/very late (>1 year) stent thrombosis, is a quite rare but serious complication that often results in acute myocardial infarction^124^. Early stent thrombosis is principally attributed to procedural and stent factors, such as stent underexpansion, large stent edge dissection and hematoma, whereas late/very late is mostly related to stent malapposition, uncovered struts, as well as neoatherosclerosis^125^. Intravascular imaging, preferred OCT, can help to detect the presence of thrombus and identify factors likely to have contributed to thrombosis.

Besides, novel hybrid imaging systems, including IVUS-NIRS, OCT-NIRF, and OCT-NIRS have also been attempted for characterizing ISR and stent thrombosis in pre-clinical and small-scale clinical studies, demonstrating high feasibility and efficacy^126–128^. Findings on these hybrid imaging modalities are likely to provide more detailed information for revealing the precise mechanisms underlying a stent failure event.

## Safety Concerns

Notwithstanding the improved stenting results and long-term benefits, intravascular imaging modalities, especially OCT, remain underutilized in real world PCI practice, in particular for ACS patients^129^. Besides the barriers of expertise requirement and increased medical cost, safety concerns might contribute to the reluctance to perform the additional procedure as well. Two randomized studies have found that the use of OCT in acute myocardial infarction patients is associated with a prolonged procedural duration and a greater volume of contrast medium, but not accompanied by the increased risk of periprocedural myocardial infarction and acute kidney injury^109,110^. However, findings of these studies are limited by the small sample size and required to be further evaluated. Performance of intravascular imaging in patients with severe hemodynamic disorder or high risk of acute kidney injury should be approached with caution, especially with the use of OCT. Another notable safety concern is that intravascular imaging may cause procedure-related complications, for instance, transient ST-elevation, coronary spasm, bradycardia, iatrogenic dissection, and stent deformation^130^. Nevertheless, intravascular imaging is considered quite safe now, as advancements in imaging technology and the standardization of clinical procedures have reduced the incidence of these complications to around 0.5%, with most cases being benign and showing no significant difference in occurrence between IVUS and OCT^130^.

## Programmatic Recommendations

Theoretically, the use of an accessible intravascular imaging modality is currently appropriate for the diagnostic evaluation and PCI guidance in all ACS patients, according to the best available evidence derived from ACS subgroup analyses of previous all-comer studies and recent trials dedicated in ACS patients^7,8,111^. The 2023 European Society of Cardiology guidelines have recommended the application of intravascular imaging to guide PCI in ACS patients (Class IIa, A)^8^. Intravascular imaging, preferably OCT, is also recommended for the identification of culprit lesions in case of angiographically ambiguous findings (Class IIb, C)^8^. Recently, the 2025 ACC/AHA/ACEP/NAEMSP/SCAI guideline for the management of ACS endorsed the use of intracoronary imaging with IVUS or OCT for procedural guidance during coronary stent implantation in the left main artery or complex lesions to reduce ischemic events (Class I, A)^9^.

Practically, intravascular imaging should be particularly applicable to ACS patients with left main or complex culprit lesions, or those with ambiguous angiographic findings. Although each modality has its specific advantages and limitations, the selection of either IVUS or OCT is left to the discretion of the cardiologists because of the generally similar outcomes following OCT-guided and IVUS-guided PCI, with the exception of angiographically ambiguous culprit lesions for which OCT is generally preferred^8^. OCT should also be more appropriate for the evaluation of stent failure and the identification of vulnerable plaques in nonculprit lesions as compared with IVUS. Of note, safety precautions must be taken in case of pre-existing patient and lesion conditions at a high predisposition of imaging-related complications, such as haemodynamic instability, highly stenotic lesions, or extremely tortuous anatomy.

Following the identification of a culprit lesion with a definite atherosclerotic cause by intravascular imaging, revascularisation should be appropriate in current clinical practice, but whether a conservative strategy with optimal medical therapy only for culprit lesions with plaque erosion with less severe residual stenosis or with a nonatherosclerotic cause without high-risk indications could be at least equally effective is required to be further confirmed by prospective, randomized, controlled trials. If accessible, the application of dedicated software for image-based virtual stenting might be helpful for PCI planning, although the utility is required to be further validated. Based on the current data presented in this review article, PCI optimization will be recommended to meet the optimal stenting results beyond the restoration of epicardial blood flow^90^. Nevertheless, the potential risks of PCI optimization procedures, such as the aggravation of microvascular obstruction and vessel wall injury should also be carefully evaluated beforehand^131,132^.

## Perspectives

In the past decade, significant research efforts have focused on advancing intravascular imaging technologies. High-definition IVUS systems, particularly those utilizing 60 MHz transducers, have significantly improved resolution and are already used in clinical practice. Experimental systems with frequencies exceeding 80 MHz show even better resolution, but face challenges with signal attenuation in blood^133^. Innovations like dual-frequency transducers and optical ultrasound detectors are under investigation to enhance image quality while maintaining deep tissue penetration. OCT has also advanced with features such as polarization-sensitive OCT and Doppler imaging, which allow better tissue differentiation and blood flow quantification, providing more detailed insights into plaque composition and vessel dynamics^134–136^. Validation of polarization-sensitive OCT in coronary artery is currently at the first-in-human stage^137^. Micro OCT is another promising development, offering ultra-high resolution (1–4 μm) for detailed imaging of endothelial cells, inflammatory markers, and atherosclerotic structures. However, its adoption is likely to be hampered by its reduced penetration depth and the large data volumes generated. Emerging molecular imaging technologies, including NIRF, FLIm, and IVPA, offer unique advantages in areas such as inflammation detection and plaque composition identification^78,138–142^. The standalone use of these modalities, as well as their integration with IVUS or OCT to simultaneously assess both morphological and molecular characteristics, holds great promise for enhancing clinical capabilities and providing more complete insights into coronary artery disease^75,138^.

Advances in AI have significantly expanded the potential for image processing and interpretation in intravascular imaging. These tasks include lumen segmentation, plaque characterization and quantification, plaque vulnerability assessment, thrombus detection, automatic stent segmentation, and stent underexpansion prediction^29,143–146^. The integration of AI can streamline the time-consuming and labor-intensive tasks of manual analysis, reduce inter-observer and intra-observer variability, and enhance real-time clinical decision-making^19,147^. However, AI in intravascular imaging still faces significant barriers to wide clinical adoption, including anatomical variability, imaging artifacts, and the necessity for rigorous validation.

Computational coronary physiology has also made strides in clinical applications, particularly in ACS. Traditional wire-based fractional flow reserve can be influenced by transient microvascular dysfunction in ACS^148^. In contrast, imaging-based computational coronary physiology combines OCT or IVUS with fluid dynamics models to assess coronary function directly based on geometry, independent of microcirculation status^149^. This offers a promising alternative for coronary physiology assessment in ACS patients, potentially allowing a “one-stop-shop” for comprehensive coronary assessment, covering anatomy, function, and plaque composition in a single imaging pullback^150^.

Despite remaining challenges, with broader clinical validation and the standardization of protocols, these advancements are expected to enhance the accuracy, efficiency, and outcomes of ACS management in the near future.

## Conclusion

The beneficial role of intravascular imaging for the diagnosis and management of ACS through direct visualisation of coronary plaques and stent struts has been increasingly recognized. Over the past decade, intravascular imaging has made significant strides, both in traditional morphological imaging and emerging functional or molecular imaging. Building on this foundation, hybrid imaging is also rapidly advancing by integrating morphological and molecular information for more precise and comprehensive assessment. The integration of AI tools further facilitates image processing and interpretation and additional evaluations beyond plaque metrics. Ongoing efforts should be made to refine the imaging techniques and devices for ease of access and to develop robust AI models with clinical acceptance for fully automated image analysis. The establishment of an efficient workflow for appropriate use of intravascular imaging, involving the standard criteria for treatment strategy selection, stent sizing, as well as PCI optimization, would also improve clinical adoption.

## Data Availability

No datasets were generated or analysed during the current study.
